# The Influence of Perceived Autonomy Support on Physical Activity Among High School Students: The Mediating Roles of Basic Psychological Needs

**DOI:** 10.3390/bs15040536

**Published:** 2025-04-16

**Authors:** Zhou Wanwan, Ahmad Zamri Khairani

**Affiliations:** 1 School of Educational Studies, Universiti Sains Malaysia, George Town 11800, Penang, Malaysia; zhouwanwan@student.usm.my; 2 Department of Physical Education, Bozhou University, Bozhou 236800, China; 3 National Higher Education Research Institute, Bayan Lepas 11900, Penang, Malaysia

**Keywords:** perceived autonomy support, basic psychological needs, physical activity, mediating effect

## Abstract

Parental and peer support has been documented as an important factor in physical activities among high school students. Likewise, coach support has an important impact on physical activity among high school students. Meanwhile, many studies show that basic psychological needs have an essential effect on perceived autonomy and physical activity. As such, this study, using self-determination theory, aimed to explore the mediating role of basic psychological needs on physical activity among high school students in China. The Sport Climate Questionnaire, Psychological Need Satisfaction in Exercise Scale—Physical Activity, and the three-item Physical Activity Rating Scale-3 were employed to gauge responses from 736 high school students (15–18 years old). A structural equation model was employed to test the mediating effect. The researchers found a significant positive effect of perceived coach support on basic psychological need satisfaction. Basic psychological need satisfaction was also found to be a significant predictor of physical activity. Surprisingly, the results showed that perceived coach support negatively influences physical activity. As such, an indirect mediating effect is established. Basic psychological needs had an indirect mediating effect and could reduce the negative effect of coach support on physical activity engagement.

## 1. Introduction

Physical activity indicates any skeletal muscle activity requiring energy expenditure (World Health Organization, 2020). It can encompass a wide range of activities in our daily lives, such as walking, running, dancing, swimming, yoga, and gardening. The global significance of physical activity cannot be overstated, given its profound impact on individuals’ and societies’ health and well-being (Cecchini & Carriedo, 2020; Ozemek et al., 2018). Undeniably, physical activity is indispensable for maintaining good health. A lack of physical activity ranks among the primary causes of chronic diseases globally, including obesity, cardiovascular ailments, and diabetes (Kerr et al., 2017). Research shows that regular and moderate physical activity can improve cardiopulmonary function, enhance metabolic processes within the body, boost immunity, and decrease the likelihood of developing diseases (Amanat et al., 2020).

Further, physical activity also helps in weight management, reducing the prevalence of obesity and protecting against diseases (Elagizi et al., 2020; Wyszyńska et al., 2020). Physical activity is also of great assistance in stabilizing mental health. The deficit of physical activity contributes to symptoms of anxiety and depression that, in turn, promote issues related to emotional well-being and stability (Felez-Nobrega et al., 2023). Research has found that physical activity is important for children’s early mental health (Agostino et al., 2023; De Greeff et al., 2018). In addition, physical activity also exerts a positive impact on cognitive function. Research has revealed that moderate physical activity can enhance the structure and function of the brain, leading to improvements in memory, concentration, and intrapersonal abilities (Erickson et al., 2009; Valkenborghs et al., 2019).

Given that long-term engagement in physical activity is influenced by motivation, understanding the role of autonomy support becomes crucial. There is increasing research on perceived autonomy support as a critical factor in promoting physical activity (Choi et al., 2017; Hu et al., 2021). Perceived autonomy support is an important predictor of physical activity because it encourages people to exercise based on their preferences, passions, and internal motivations rather than outside demands (Haerens et al., 2015). When people feel empowered to make their own decisions, they are more likely to participate in and sustain behaviors encouraging well-being, including regular physical activity (Ryan & Patrick, 2009). Furthermore, autonomy is associated with better mental health, which might help people to want to work out more (Deci & Ryan, 1987). Since it reduces stress and increases satisfaction, people are more likely to repeat an activity when they feel empowered to make their own judgments (Vansteenkiste et al., 2005).

It is crucial to note that physical activity is strongly associated with a person’s satisfaction with their fundamental psychological needs and perceived autonomy support (Ryan & Deci, 2017). Three requirements—autonomy, competence, and relatedness—are commonly employed to characterize basic psychological needs (Ryan & Deci, 2017). Correspondingly, Vansteenkiste et al. (2020) describe relatedness as warmth, bonding, and compassion, whereas autonomy is the sensation of volition and willingness. Conversely, competence means using and expanding abilities and knowledge through experiences and activities. These three needs are essential to promote intrinsic motivation and sustained engagement in physical activity. When these demands are satisfied, individuals engage in physical activity more willingly and frequently, which enhances their overall health and well-being.

The literature shows that many studies have examined the relationship between parental support and physical activity (Hui et al., 2022). In many studies, researchers focus on individuals who provide support, such as family, peers, etc. To illustrate, a review by Beets et al. (2010) concluded that parental social support, both tangible and intangible, has positive associations with youth physical activity-related behaviors. According to Gustafson and Rhodes (2006), solid parental support can be seen through their encouragement, involvement, and facilitation. In addition, Pyper et al. (2016) observed that students’ performance was enhanced when parents were supportive of their physical activity. Apart from parents, peers were also influential in students’ engagement in physical activity. Meanwhile, peer social support is associated with positive physical activity, where adolescents who received more peer support were more physically active than those receiving less support (Haidar et al., 2019). In short, parents who show excitement and verbally support their children foster confidence and a nurturing atmosphere that encourages participation in physical activities. Meanwhile, having peers who are also physically active can be motivating because the students feel more supported and experience a bond with their peers.

While the influence of parents and peers has been widely studied, coaches’ autonomy support remains a neglected area of research. In this research, coach refers to an educator or professional who is responsible for guiding and managing the sports training and competition of high school students, usually playing a role in school physical education courses, school team training, or extracurricular physical activity. In particular, there is a limited understanding of how coaches’ provision of autonomy support impacts students’ motivational outcomes and engagement in physical activities. Coaches can have both positive and negative influences on physical activity (Erickson et al., 2009). As such, we hypothesize that perceived autonomy support from the coach positively and significantly influences physical activity (Ha1).

There are lots of studies on basic psychological need satisfaction and physical activity. For example, research by L. H. Zhou et al. (2019) showed that support from parents, teachers, and peers positively impacts Chinese primary school students’ psychological need satisfaction. Similarly, Li et al. (2019) found a significant influence of autonomy-supportive teaching on basic psychological needs among secondary school students in China. As such, the following hypothesis was formulated: perceived autonomy support positively influences satisfaction with basic psychological needs (Ha2).

The influence of basic psychological needs on physical activities is relatively less well explored, even though the two constructs are significantly related (Dunton et al., 2023; Gurleyik et al., 2022; Stults-Kolehmainen, 2023). It should be noted that when basic psychological needs are fulfilled, individuals are more likely to engage in and sustain physical activities due to increased intrinsic motivation, enjoyment, and personal investment (Westerskov Dalgas et al., 2024). When people feel autonomous in their choices of physical activity, they are more likely to enjoy the activity and stick with it in the long term (Thal et al., 2024). Autonomy fosters intrinsic motivation, making individuals more likely to engage in physical activities for the pleasure and satisfaction they derive rather than external pressures (Mossman et al., 2024). Likewise, when people feel competent in their physical activities, they are more likely to experience enjoyment and continue exercising (Southcott & Jooste, 2024). Meanwhile, regarding relatedness, a person who engages in physical activities and forms friendships with other participants is more inclined to continue attending classes due to the sense of connection (Eubank & DeVita, 2024). Based on these arguments, we propose the following hypothesis: basic psychological needs mediate the relationship between perceived autonomy support and physical activity (Ha3).

## 2. Methods

### 2.1. Study Design

The present study was conducted using a correlational research design and online surveys. Before beginning the study, participants were informed that the questionnaire would be entirely anonymous, that the data would only be used for academic research, and that only the researchers would have access to it. In addition, online informed consent was acquired.

### 2.2. Setting

Participants were from six public high schools in urban areas of Anhui Province. They were between 15 and 18 years old. Interested students and their parents submitted signed consent forms before data collection. Schools were selected based on the diversity of student socioeconomic backgrounds to minimize selection bias. The data were collected between June and August 2023 via a Chinese online survey platform (Questionnaire Star).

### 2.3. Sampling and Participants

Participants were recruited through purposive sampling from six public high schools in urban areas of Anhui Province. The study was granted an exemption from full IRB review because it involved minimal risk and collected anonymous survey data. The researcher first obtained permission from each school’s administrative board. Eligible students (aged 15–18) were invited through classroom announcements and informational flyers. Interested students and their parents submitted signed consent forms before data collection. Schools were selected based on the diversity of student socioeconomic backgrounds to minimize selection bias. Participation was entirely voluntary, with no academic incentives or penalties. Students could decline involvement without repercussions, as emphasized in both oral and written communications.

### 2.4. Outcome Measures

The 6-item single dimension short form Chinese translation of the Sport Climate Questionnaire (SCQ) by Lim and Wang (2009), was used to measure perceived autonomy support. The Chinese translation has been verified (Ding & Mao, 2014; Hui et al., 2022). The questionnaire usually identifies a particular coach or individual in similar roles in a sport or physical activity. Evidence of reliability and convergent validity of the measurement is reported in Table 1. Meanwhile, the satisfaction of the BPN was measured using a 20-item Psychological Need Satisfaction in Exercise Scale—Physical Activity (PNSES—PA) (Vlachopoulos & Michailidou, 2006). The Chinese translation has been verified (Ding & Mao, 2014). The PNSES-PA demonstrated high reliability (Cronbach’s α = 0.947) in this study. The instrument includes three dimensions, namely (1) autonomy, (2) competence, and (3) relatedness. On the other hand, physical activity is measured using the 3-item Physical Activity Rating Scale-3 (PARS-3) (Liang, 1994). This scale is divided into intensity, time, and frequency, with each dimension having five grades; intensity and frequency from grades 1 to 5 are recorded as 1 to 5 points, and time from grades 1 to 5 is recorded as 0 to 4 points. The score is calculated using the following equation: PA score = PA intensity score × (PA duration score − 1) × PA frequency score). The PA score was used to categorize the PA level into three groups: low ≤ 19 points; moderate = 20 to 42 points; and high ≥ 43 points. This scale has been widely used in sports science research in China and was put into use in this study without revisions (Hui et al., 2022).

### 2.5. Bias

In the context of PLS-SEM, CMB refers to a phenomenon induced by the measurement method employed in the SEM study, rather than by the causal network within the model under investigation (Kock, 2017). Kock and Gaskins (2014) highlighted that CMB can be identified and evaluated based on complete collinearity. Kock (2017) highlighted that CMB can be detected through a thorough collinearity assessment method based on variance inflation factors. A VIF of 5 or above indicates the presence of collinearity (Hair et al., 2019). Table 2 shows the variance inflation factor (VIF) values associated with our study. All VIF values are within the critical range. Therefore, our study did not show significant common methodological bias problems.

### 2.6. Sample Size

In determining the sample size, the researchers followed the guidelines by Hair et al. (2019): if the total number of constructs in the model was less than or equal to 7, the minimum sample size was 300; if the total number of constructs exceeded 7, the minimum sample size was 500. Therefore, the sample size in this study was 736.

### 2.7. Statistical Analysis

Data were analyzed using the IBM SPSS 28.0 and SmartPLS 4.0 programs. Descriptive statistics and reliability analyses were conducted in SPSS 28.0, while structural equation modeling (SEM) was performed using SmartPLS 4.0. SPSS 28.0 provided basic information about the measurement, especially the evidence of both construct and discriminant validity. Separately, SmartPLS 4.0 was used to examine the structural relationship between the variables and the mediation analysis.

## 3. Results

Table 1 presents evidence of the measurement’s convergent validity and reliability using these instruments. All results are within the prescribed scope.

**Figure 1:**
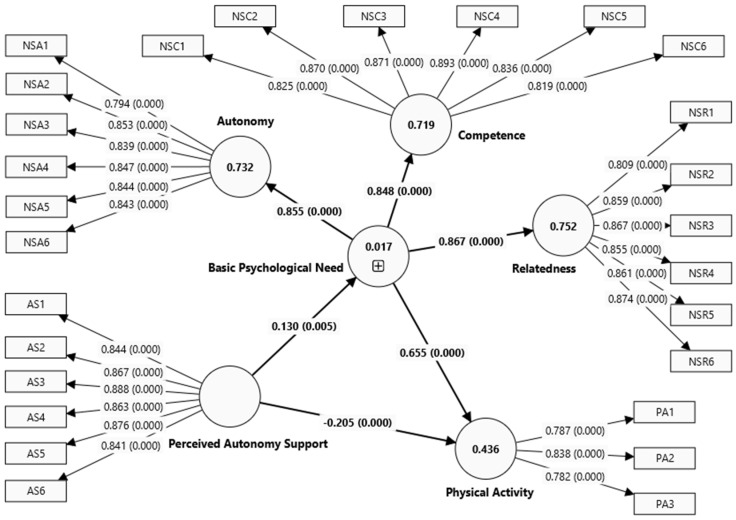
Structural model.

Table 3 shows descriptive statistics of the minimum score, maximum score, mean, standard deviation (SD), skewness, and kurtosis of the measurement. All data were collected using instruments with a five-point Likert-like rating scale. No factor loading coefficients met the criteria for deletion. Meanwhile, Figure 1 shows the structural model of the link between perceived autonomy support, basic psychological needs, and physical activity. According to Kim (2013) and Mishra et al. (2019), for sample sizes larger than 300, a guideline for normality is that the absolute value of skewness should be less than or equal to 2, or the absolute value of kurtosis should be less than or equal to 4. Hence, it can be inferred that the data gathered in this study do not deviate from the normal distribution, rendering them suitable for analysis using PLS-SEM.

Henseler et al. (2015) suggest that the threshold for HTMT should be lower than 0.90. Table 4 presents the HTMT values observed in this study, all of which are below the critical threshold of 0.655. Hence, this indicates that the structural model of this study has conceptually distinct structures.

In the mediation analysis shown in Table 5, all the direct effects are significant. To illustrate, a higher level of perceived autonomy support leads to a higher level of basic psychological needs (beta = 0.130, *p* = 0.005), leading to higher engagement in physical activity (beta = 0.655, *p* < 0.001). As for the direct effect, we reported a significant but negative influence of perceived autonomy support on physical activity (beta = −0.205, *p* < 0.001). Partial mediation is established based on indirect and direct effects. Another observation from this result was that there is a competitive mediation, since the indirect effect is positive while the direct impact of perceived autonomy support on physical activity is negative.

## 4. Discussion

Although various studies show the positive influence of coaches on physical activity (Fenton et al., 2014; Reynders et al., 2019), findings from the present study report otherwise. There is a valid explanation for why this result was not expected. Research in Western contexts often emphasizes the intrinsic motivation promoted by autonomy support, whereas in some East Asian educational settings, autonomy support from authority figures (e.g., coaches) may still be perceived as controlling rather than empowering. In collectivist cultures, excessive emphasis on individual autonomy may trigger anxiety that conflicts with social expectations, thereby inhibiting movement participation (Chirkov et al., 2003). A coach who places too much emphasis on the perfection of skills rather than enjoyment and personal growth might hinder students’ engagement in physical activity (Standage et al., 2003). This can create high levels of pressure, which may make some students feel anxious or discouraged, potentially leading them to lose interest in physical activity. One possible explanation for this is because of the culture of China. Chinese culture traditionally emphasizes discipline, hard work, and the pursuit of excellence (Wu, 2016). This cultural influence often translates into a coaching style that prioritizes rigorous training, repetition, and mastery of technique over other aspects like creativity or playfulness (Morris et al., 2005). Overemphasizing discipline has also resulted in many Chinese coaches following an authoritarian style that prioritizes discipline and obedience. (Morris et al., 2005). This style is often associated with intensive drills and a strong emphasis on technical perfection, as opposed to more collaborative or flexible approaches. Therefore, this approach might make students shy away from engaging in physical activity, especially at high school levels, since they prefer enjoyment, team games, outdoor lessons, etc. (Y. Zhou & Wang, 2019), which commonly do not involve much emphasis on skill drilling.

Another potential explanation for coaches’ negative influence on physical activity is insufficient attention being paid to individual needs during physical activity sessions (Amorose & Anderson-Butcher, 2007). It should be noted that students vary widely in their athletic abilities, physical conditions, and emotional needs (Haegele & Hodge, 2016). Coaches who fail to tailor activities or provide individualized support may unintentionally push students beyond their comfort capability (Bartholomew et al., 2011). It should be noted that students have unique physical conditions and limitations that affect their performance. For example, while strength training can be beneficial, intense activities such as weightlifting are often not encouraged for younger students (Lloyd et al., 2014). Their bodies are still developing, and improper technique or overloading can lead to injuries (DiFiori et al., 2014). As such, by adapting activities to students’ physical state, coaches can help to prevent overtraining and avoid asking students to engage in activities that are not suitable for their physical state.

In contrast to physical activity, the researchers found that coach autonomy support positively influences basic psychological need satisfaction. Autonomy and relatedness mitigated the negative effect more effectively than competence. This finding is in line with past studies such as those by Bolier et al. (2013), Franco and Coterón (2017), and Yu et al. (2020). It is widely known that discipline encourages consistent, focused practice, which is essential for skill development and improvement (Ryan & Deci, 2020). When students see progress and gain mastery over the activities they are engaged in, it boosts their sense of competence (Deci & Ryan, 2000). Knowing that they can meet challenging demands enhances their confidence and satisfaction with their abilities (Schunk, 1991). In addition, it should be noted that discipline does not have to mean rigidity. When students understand the purpose behind disciplined routines and have input in setting goals or planning aspects of their activities, it nurtures their sense of autonomy (Jang et al., 2010). Coaches who communicate the “why” behind discipline foster buy-in and empower students as they actively commit to their goals (Delrue et al., 2019).

Comparatively, shared commitment to discipline fosters a sense of camaraderie and connection within a team. When students see coaches and teammates dedicating themselves to disciplined routines, they build trust and unity. Students feel they belong to a group that values hard work, creating a supportive environment where they feel understood and connected. However, the researchers found that basic psychological needs positively and significantly influence physical activity. When the students’ psychological needs are fulfilled, they tend to engage more in physical activity. Regarding autonomy, when students feel they have control over their choices in physical activity, they are more motivated to participate (Huéscar Hernández et al., 2020; How et al., 2013). This sense of freedom leads to a more enjoyable and personally meaningful experience, encouraging them to stick with it. Regarding competence, feeling capable and effective in physical activities promotes satisfaction and confidence (Haitao, 2011; Jackson et al., 2013). When students see progress, such as improved fitness or skill mastery, it reinforces their desire to continue. Meeting skill-based challenges or achieving fitness goals boosts self-efficacy, making them more likely to stay active and pursue further improvements.

Finally, the positive influence of basic psychological needs that significantly influence physical activity may also be explained by relatedness. Physical activity often has a social component, whether working out with friends, joining a sports team, or being supported by a coach. When students feel connected and supported, it increases their enjoyment and sense of belonging. This supportive environment fosters a commitment to regular physical activity and can make it a positive, long-term habit (Khan et al., 2020). Relatedness not only increases participation and improves the quality of direct physical improvement activities through social interaction, emotional support, and psychological identification, but also indirectly improves the multi-dimensional positive cycle of health and activity (Gunnell et al., 2014).

Based on the results for all hypotheses, the researchers conclude that basic psychological needs indirectly mediate the relationship between coach autonomy support and physical activity among high school students. That is, the fulfillment of basic psychological needs (autonomy, competence, and relatedness) explains some of the negative effect that a coach’s autonomy-supportive style has on an athlete’s engagement in physical activity. More specifically, when coaches support autonomy through discipline, students feel more self-motivated, thus helping to meet athletes’ basic psychological needs through autonomy, competence, and relatedness. Students are more likely to enjoy, commit to, and engage in physical activity when these needs are met. Nevertheless, it should be noted that since this mediation is partial, other factors (like personal motivation, physical goals, enjoyment of the sport, etc.) also contribute to the increase in physical activity beyond need satisfaction.

Modeling the relationships among coach autonomy support, basic psychological needs, and physical activity is significant because it explains how these elements interact to promote sustained motivation, engagement, and well-being in athletes. By creating such a model, coaches, teachers, and trainers can identify and implement effective strategies to foster long-term physical activity. By modeling how basic psychological needs (autonomy, competence, and relatedness) mediate the relationship between coach autonomy support and physical activity, researchers and coaches can see how fulfilling these needs leads to greater motivation and enjoyment. Again, the modeling shows that autonomy support is practical, not only because it directly impacts physical activity but also because it fulfills fundamental psychological needs. This understanding enables coaches to focus on strategies that meet these needs, knowing that they lead to increased motivation and positive outcomes. Physical activity engagement often declines when motivation is extrinsic (driven by rewards or external pressure). A model that links coach autonomy support and need fulfillment with increased physical activity provides insights into fostering intrinsic motivation, which is more likely to lead to sustained engagement. Finally, strategies to improve engagement include reducing excessive performance pressure, incorporating student input into training plans, and fostering a more autonomy-supportive climate through positive feedback.

Nevertheless, the present study has several limitations. First, individual differences among students—notably in personality traits, motivation levels, and life experiences—may influence their responses to autonomy support. Second, methodological constraints such as sampling bias, reliance on self-reported data, and potential social desirability bias could affect the validity of the findings. Given that this research employed purposive sampling, the researcher acknowledges that the results may lack generalizability to broader populations. The model may oversimplify these individual differences if all athletes react similarly to discipline-oriented coaches. However, factors like age, gender, cultural background, and personal motivation can alter how needs are fulfilled or prioritized, making it challenging to apply this one-size-fits-all model. Secondly, coaching styles and relationships with students are dynamic. Thus, autonomy support can vary based on the students’ progress, team goals, and competitive pressures. A static model such as in this study may fail to capture these dynamics, making it less adaptable to real-life coaching situations. Thirdly, we admit that psychological needs are difficult to measure. The self-reported measures used in this study may be biased or inconsistent, limiting the model’s accuracy. Moreover, the extent to which these needs are met can fluctuate over time and vary by individual, making it hard to capture these changes. This study highlights the complex role of autonomy support in physical activity engagement, particularly within structured coaching environments. Future research should explore longitudinal effects and consider qualitative methods to better understand students’ perceptions of autonomy support.

## 5. Conclusions

The present study aimed to develop a model that relates coach autonomy support, basic psychological needs, and physical activity and examines the mediating effect of basic psychological needs. As a result, the researchers found that coach autonomy support negatively influences physical activity engagement among Chinese high school students but positively influences basic psychological needs. Conversely, it is worth noting that basic psychological needs positively and significantly influence physical activity. As such, basic psychological needs indirectly mediate the relationship between coach autonomy support and physical activity. The researchers’ findings indicate that perceived coach autonomy support negatively predicts physical activity (*β* = −0.205, *p* < 0.001), with basic psychological needs demonstrating partial mediation (*R*^2^ = 0.436). This highlights the complex interplay between autonomy support and motivation. Furthermore, the results suggest that the motivational consequences of autonomy support may vary across cultural and contextual settings, challenging the cross-cultural applicability of self-determination theory in sports coaching. Given these findings, the researchers suggest that specific strategies could be applied to encourage physical activity among high school students, including the following:

1. Incorporating more student input into training decisions to balance discipline with autonomy;
2. Using positive reinforcement rather than excessive correction to enhance competence and motivation;
3. Providing a supportive rather than authoritarian environment to reduce the pressure associated with physical activity.

Future research should explore longitudinal changes in autonomy support’s effects, consider qualitative approaches to better understand students’ perceptions, and examine cross-cultural variations in coaching styles.

## Figures and Tables

**Table 1:** Factor loading, reliability indices, and average variance extracted (AVE).

Variable	Items	Factor Loading	Reliability			AVE
			Cronbach’s Alpha (α)	CR (rho_a)	CR (rho_c)	
Autonomy Support	AS1	0.876	0.932	0.950	0.942	0.729
	AS2	0.892				
	AS3	0.889				
	AS4	0.822				
	AS5	0.863				
	AS6	0.776				
Autonomy	NSA1	0.794	0.914	0.915	0.933	0.700
	NSA2	0.853				
	NSA3	0.839				
	NSA4	0.847				
	NSA5	0.844				
	NSA6	0.843				
Competence	NSC1	0.825	0.925	0.926	0.941	0.727
	NSC2	0.870				
	NSC3	0.871				
	NSC4	0.893				
	NSC5	0.836				
	NSC6	0.819				
Relatedness	NSR1	0.809	0.926	0.927	0.942	0.730
	NSR2	0.859				
	NSR3	0.867				
	NSR4	0.855				
	NSR5	0.861				
	NSR6	0.874				
Physical Activity	PA1	0.778	0.926	0.927	0.942	0.730
	PA2	0.837				
	PA3	0.791				

**Table 2:** VIF index of all items in this research.

Structures	Items	VIF Values	Structures	Items	VIF Values
BPN	AU1	2.141	PAS	PAS1	2.623
	AU2	2.733		PAS2	2.906
	AU3	2.555		PAS3	3.204
	AU4	2.603		PAS4	3.097
	AU5	2.559		PAS5	3.157
	AU6	2.569		PAS6	2.759
	COM1	2.447	PA	PA1	1.504
	COM2	3.023		PA2	1.558
	COM3	3.076		PA3	1.306
	COM4	3.325			
	COM5	2.506			
	COM6	2.254			

Note: VIF = variance inflation factor; BPN = satisfaction of basic psychological needs, including AU1–RE6; PAS = perceived autonomy support, including PAS1–PAS6.

**Table 3:** Descriptive statistics.

Constructs	Min	Max	Mean	SD	Skewness		Kurtosis	
					Statistic	Std.Erorr	Statistic	Std.Error
Perceived Autonomy Support	6	30	21.89	5.634	−0.264	0.09	−0.471	0.18
Satisfaction of the Basic Psychological Needs	23	90	60.92	13.584	−0.103	0.09	−0.133	0.18
PA	3	15	9.5	2.535	−0.245	0.09	−0.138	0.18

**Table 4:** Discriminant validity.

	Perceived Autonomy Support	Physical Activity
Autonomy	0.143	0.603
Competence	0.082	0.702
Relatedness	0.135	0.655

**Table 5:** Mediation analysis.

	Original Sample	Sample Mean	95% Confidence Interval	SD	T Statistics	*p* Values
Perceived Autonomy Support → Basic Psychological Need → Physical Activity	0.085	0.086	[0.019; 0.013]	0.031	2.757	0.006

## Data Availability

The data presented in this study are available upon request from the corresponding author.
